# Lipid-dependent growth of *Malassezia* spp. in defined medium with single fatty acids

**DOI:** 10.1093/femsyr/foaf043

**Published:** 2025-08-21

**Authors:** Janny Liebregts, Lars van der Velden, Angie Lorena Fonseca-Fernández, Adriana Marcela Celis Ramírez, Hans de Cock

**Affiliations:** Microbiology, Department of Biology, Utrecht University, 3584CH Utrecht, The Netherlands; Microbiology, Department of Biology, Utrecht University, 3584CH Utrecht, The Netherlands; Grupo de Investigación Celular y Molecular de Microorganismos Patógenos (CeMoP), Department of Biological Sciences, Universidad de los Andes, 111711 Bogotá, Colombia; Grupo de Investigación Celular y Molecular de Microorganismos Patógenos (CeMoP), Department of Biological Sciences, Universidad de los Andes, 111711 Bogotá, Colombia; Microbiology, Department of Biology, Utrecht University, 3584CH Utrecht, The Netherlands

**Keywords:** *Malassezia* spp, lipid-dependent growth, defined medium, free-fatty acid, lipidome

## Abstract

Details on fatty acid and lipid metabolism in *Malassezia* spp. are limited, amongst others, because efficient growth of *Malassezia* spp. in defined media with free fatty acids has not yet been described. Here, we describe a culturing method in a defined medium in which lipid-dependent growth of *Malassezia* spp. can be studied. We observed efficient growth of *Malassezia furfur* and *Malassezia pachydermatis* in liquid minimal medium supplemented with palmitic acid in the presence of NP-40 Tergitol™. We introduced a 3-day fatty acid-starvation phase to reduce residual growth due to the carry-over of lipids from rich media. The *Malassezia* spp. studied remained viable longer in liquid media lacking fatty acids and detergents, as described previously for a *Saccharomyces cerevisiae fas1* mutant. This suggests that *Malassezia* spp. might have developed mechanisms to survive periods of fatty acid starvation. We compared the lipidome of both *Malassezia* species grown in mDixon or a defined medium with NP-40 Tergitol™ supplemented with either palmitate and/or oleic acid, or ox bile. Remarkably, the lipidome of mDixon grown cells is enriched in lipid species associated with lipid droplets. *Malassezia* spp. adapt their lipid composition after growth in a defined medium, and a subset of novel lipid species was identified.

## Teaser

Growth of two Malassezia yeast species was most efficient in a new defined culture medium using either palmitic or oleic acid with NP-40 Tergitol™ allowing new studies on lipid metabolism.

## Introduction

*Malassezia* spp. are lipophilic yeasts dependent on fatty acids (FAs) in their environment for growth, e.g. present in lipids or as free fatty acids (FFAs) on the skin of mammals (Gupta et al. 2004, Rhimi et al. 2020). *Malassezia* spp. are considered commensals but can also be associated with several infections (Rhimi et al. 2020) such as seborrheic dermatitis, atopic dermatitis, pityriasis versicolor (PV), and *Malassezia* folliculitis (Harada et al. 2015). Furthermore, *Malassezia* spp. have been associated with inflammatory bowel disease (Limon et al. 2019), fungemia (Rhimi et al. 2020), as well as with pancreatic cancer (Aykut et al. 2019), but this latter study was recently challenged (Fletcher et al. 2023). A variety of *Malassezia* spp., to date at least 21 (Naik et al. 2025), have been identified, but it is anticipated that more species remain to be discovered, given that research has primarily focused on human subjects and domesticated animals (Cabañes et al. 2016, Guillot and Bond 2020).

The lipophilic nature of *Malassezia* is due to its inability to synthesize saturated FAs *de novo*, caused by the absence of the cytosolic FA synthase genes (*fas*) as revealed by genome sequencing (Xu et al. 2007, Barsh et al. 2015, Triana et al. 2015). Therefore, *Malassezia* spp. are cultured in lipid-rich media, e.g. mDixon, which contain a variety of lipid species amongst others present in ox bile (Hu et al. 2018). In addition, Tween 40 and purified oleic acid (OA) are major FA sources in mDixon for *Malassezia* spp. FAs from lipids or Tween are released after hydrolysis by secreted lipases (Park et al. 2021). After the uptake of an FFA and concomitant activation with CoA via long-chain acyl-CoA synthases (Tenagy et al. 2019), FA-CoA species can be further modified in length and saturation and incorporated into lipids. FAs can furthermore be stored as triglycerides (TGs) and sterol esters in lipid droplets or degraded by beta-oxidation in peroxisomes. These processes have been extensively studied in *Saccharomyces cerevisiae*; see for a recent review (Wang et al. 2024). Previously, *Malassezia* spp. were cultured in a defined medium with FFAs in the presence of the detergent Brij-58, but growth appeared inefficient (Triana et al. 2017). In contrast, efficient growth of a *fas1* mutant of *S. cerevisiae* in YEPD medium with FAs and Brij-58—with an FA preference for myristate over palmitate and stearic acid (SA)—was observed (Schweizer and Bolling 1970). Additionally, efficient growth of the *S. cerevisiae fas* mutant in a defined medium with FFA in NP-40 Tergitol™ was observed (Henry 1973). Remarkably, the *S. cerevisiae fas* mutant lost viability very fast upon culturing in minimal medium lacking FAs and NP-40 Tergitol™, and approximately, two logs decrease of viability was observed over 8 h of culturing (Henry 1973).

In this paper, we developed a culturing method in a defined medium for *Malassezia* spp. in order to open up the possibilities for studies on lipid metabolism. We observed efficient growth of *Malassezia furfur* and *Malassezia pachydermatis* in liquid minimal medium supplemented with FFAs in the presence of NP-40 Tergitol™. Both *Malassezia* spp. preferred palmitic acid (PA) and hardly grew with myristic acid (MA) in contrast to *S. cerevisiae fas* mutant in these culturing conditions. Interestingly, we observed that the *Malassezia* spp. studied remained viable longer in liquid media lacking FAs and detergents as compared to as previously reported for a *S. cervisiae fas* mutant. Remarkably, the lipidome of *Malassezia* spp. grown in mDixon is enriched in lipids associated with lipid droplets in contrast to the lipidome of cells grown in a defined medium with NP-40 Tergitol™ in the presence of either ox bile or FFAs. Each of the *Malassezia* spp. adapts their lipid composition differently after growth in defined medium in the presence of specific FFA donors as revealed by lipidomic analysis.

## Materials and methods

### Fungal strains

*Malassezia furfur* CBS 1878, *M. pachydermatis* CBS 1879, and *M. sympodialis* CBS 7222 were obtained from the Westerdijk Fungal Biodiversity Institute, Utrecht, the Netherlands. The *S. cerevisiae* WT and *fas* knock-out strain BY.PK1238_KO was kindly provided by Fischer and colleagues (Institute of Organic Chemistry and Chemical Biology, Goethe University Frankfurt, Frankfurt am Main, Germany) and described previously (Fischer et al. 2020). The *S. cerevisiae* wild type (WT) strain is the BY.PK1238_KO with the *fas* gene reintroduced. Table 1 shows the strains used and their genotype.

**Table 1.** Overview of strains used in this research.

Fungal strain	Genotype	Auxotrophic requirements	Reference
*Malassezia furfur* CBS 1878	–	–	Westerdijk Institute, Utrecht, The Netherlands
*Malassezia pachydermatis* CBS 1879	–	–	Westerdijk Institute, Utrecht, The Netherlands
*Saccharomyces cerevisiae* WT	*fas*1/*fas*2	20 mg l^−1^ ura; 20 mg l^−1^ met; 50 mg l^−1^ lys	Fischer et al. (2020)
*Saccharomyces cerevisiae* BY.PK1238_KO	Δ*fas*1/Δ*fas*2	20 mg l^−1^ ura; 20 mg l^−1^ met; 50 mg l^−1^ lys; 20 mg l^−1^ his; 60 mg l^−1^ leu	Fischer et al. (2020)

ura: uracil, met: methionine, lys: lysine, his: histidine, leu: leucine

### Media and culturing conditions

*Malassezia* spp. were stored at –80°C in skim milk and used to inoculate modified Dixon (mDixon) agar plates [36-g l^−1^ mycosel agar (BBL), 20-g l^−1^ ox bile purified (Fluka/Merck), 36-g l^−1^ malt extract (Gibco), 2-ml l^−1^ glycerol (Honeywell), 2-ml l^−1^ OA (Fluka analytical), 10-ml l^−1^ Tween 40 (MERCK-Schuchardt), 0.5-g l^−1^ chloramphenicol ≥ 98% crystalline (Sigma Life Sciences). Plates were incubated at 33°C for 7 days in an incubator at high humidity and re-streaked onto new mDixon agar every week under the same conditions. After ∼6 weeks, fresh cultures were prepared from −80°C.

Two *S. cerevisiae* strains were used: the *fas*1/*fas*2 knock-out strain BY.PK1238_KO and the *S. cerevisiae* WT, which is the BY.PK1238_KO strain containing a plasmid with the *fas1 and fas2* genes. The *S. cerevisiae* WT strain was cultivated on modified YPD plates [50-g l^−1^ YPD broth (Sigma-Aldrich), 15-g/L bacteriological agar (Oxoid), 50-mg l^−1^ MA (Sigma-Aldrich), 50-mg l^−1^ PA (Avocado Research Chemicals ltd.), 50-mg l^−1^ SA (Sigma-Aldrich), 1% Tween-20 w/v (Acros organics), 20-mg l^−1^ uracil (Sigma), 20-mg l^−1^ methionine (Sigma), 50-mg l^−1^ lysine (Sigma-Aldrich), and 200-µg l^−1^ geneticin disulfate (G418) (Gibco)]. The plates were then incubated at 37°C for 3–5 days. For growth in *Malassezia* minimal media (MMM), this four-fold concentrated medium was supplemented with histidine (Sigma), leucine (Sigma), uracil (Sigma), and geneticin disulfate (Fischer et al. 2020). Composition of MMM supplemented with FFA and 1% NP-40 Tergitol™ and subsequent growth in 96 well plates in the Synergy H1 (Agilent) was performed in the same way as described for the *Malassezia* spp. at 33°C.

### Growth in defined medium

*Malassezia* spp. were cultured on mDixon plates for 7 days in a 33°C incubator with high humidity. Subsequently, three scoops of around 5-mm colonies were solubilized in 5-ml Milli-Q water via the sides of a 15-ml tube just above the water surface to prevent aggregation of cells. FA-starved cultures were made by adding 3-ml *Malassezia* cells in water to 27-ml MMM containing [10-ml l^−1^ K-buffer (200-g l^−1^ K_2_PO_4_ + 145-g l^−1^ KH_2_PO_4_), 20-ml l^−1^ M-N buffer (30-g l^−1^ MgSO_4_ · 7H_2_O + 15 g l^−1^ NaCl), 1-ml l^−1^ 1% CaCl_2_•2H_2_O w/v, 10-ml l^−1^ 20% glucose w/v, 10-ml l^−1^ 0.001% FeSO_4_ w/v, 5-ml l^−1^ Spore elements (100-mg l^−1^ ZnSO_4_ · 7H_2_O, 100-mg l^−1^ CuSO_4_ · 7H_2_O 100-mg l^−1^ H_3_BO_3_, 100-mg l^−1^ MnSO_4_ · H_2_O, 100-mg l^−1^ Na_2_MoO_4_ · H_2_O, and 2.5-ml l^−1^ 20% NH_4_NO_3_ w/v)]. The minimal medium buffers were sterilized by using 0.2-µm filters (Sarstedt). Cells (∼5*10^6^ cells/ml) were incubated for 3 days in an Erlenmeyer at 33°C at 180 rpm. Subsequently, wells of a 96 F bottom well plate (Costar) were filled with 37.5 μl 4x MMM, 82.5 μl Milli-Q and 15 μl 10x FFA (5 mM + 10% NP-40 Tergitol™) or 10% NP-40 Tergitol™ (control) and finally 15 μl FA-starved cells. For growth in defined medium with ox bile and 1% NP-40 Tergitol™, 15 μl of a 10-fold concentrated stock of 20-g l^−1^ pure ox bile (CarlRoth) or an ox bile mix (20-g l^−1^ ox bile, 2-ml l^−1^ OA, and 10-ml l^−1^ Tween 40) was used as FA source. To minimize sample evaporation, the wells on the outer edge of the plate were filled with 200-μl Milli-Q water. The well plate was closed with a lid and placed in the Synergy H1 (Agilent®). Growth was followed by determining the OD600 value which is the optical density at 600 nm (absorbance). OD600 values were measured every hour for 72 h at a temperature of 33°C with a 1°C gradient above the lid to reduce condensation. The plate was shaken in a continuous double orbital mode at 282 rpm. For control, wells were prepared for each medium source lacking the cells, and OD600 values from these control wells were subtracted from the measurements with cells, to correct for background signals attributed to both the FA source and the medium. Similar conditions were used for growth in Tween at a final concentration of 0.1% (v/v) in the absence or presence of 1% NP-40 Tergitol™. Experiments were conducted in triplicate per plate and repeated at least three times. Similar experiments were performed with the *S. cerevisiae* strains, but, in that case, 4x MMM was supplemented with the respective amino acids, uracil (Table 1), and geneticin disulfate (G418) as described (Fischer et al. 2020). All other conditions of the liquid growth experiments in the 96-well plates were performed identically. The cultures in the 96-well plates were analyzed regularly using the Zeiss Axiovert 25 microscope.

### Preparation of FA and Tween stock solutions

All FAs were used at a final concentration of 0.5 mM and added from a 5-mM stock solution in deionized water containing 10% NP-40 Tergitol™ (Sigma-Aldrich). FAs used were lauric acid (LA) (Sigma-Aldrich), MA (Sigma), PA (Avocado Research Chemicals ltd.), SA (Sigma-Aldrich), OA (Fluka), arachidic acid (AA) (Sigma), behenic acid (BA) (Sigma), and lignoceric acid (LCA) (Sigma). Furthermore, 10-fold concentrated stock of ox bile (20 g l^−1^; Carlroth) or ox bile mix with an additional 20-ml l^−1^ OA and Tween-40 (10 ml l^−1^; Acros organics) was prepared. All FA stocks were shortly heated in a microwave at 750 W, swiveling the stocks every 15 s until dissolved. When dissolved, all stocks were filter sterilized using 0.45-µm filters (Sarstedt). Before use, saturated FA stocks required slight heating again in a microwave at 750 W for 15 s to dissolve the formed crystals. FA stocks were of high purity >95%.

1% Tween (v/v) stocks were prepared using Tween 20 (Acros organics), Tween 40 (MERCK-Schuchardt), Tween 60 (Sigma), and Tween 80 (Sigma). From each Tween stock, two stock solutions were prepared, one with and one without 10% NP-40 Tergitol™, and the final concentrations used in culturing experiments were 0.1% Tween (v/v) and 1% NP-40 Tergitol™(v/v). All Tween stocks were shortly heated using a microwave at 750 W, swiveling the stocks every 15 s until dissolved. When dissolved, all stocks were filter sterilized using 0.45-µm filters (Sarstedt), and once dissolved, no further heating was required. The fatty acid methyl esters (FAME) composition of Tween 20, 40, 60, and 80 was determined using GC analysis (Bao et al. 2021), which revealed that all Tween types were not pure, and especially Tween 20 contains various FA (Supplementary Fig. S1).

### Detection of lipid droplets

Cells were grown until an optical density at 600 nm (OD600) of 1.0 or higher. Cultures were divided evenly into 15-ml conical bottom tubes and centrifuged at 5000 g for 5 min using the 5920R Eppendorf centrifuge. After centrifugation, supernatants were removed, and cell pellets were washed three times with 5-ml saline solution. Cells were then resuspended in 1.5-ml saline solution, and 1 ml of this suspension was transferred to a dedicated Eppendorf tube. For fluorescence staining, 10 μl of 1-g l^−1^ Calcofluor White (Sigma Aldrich) and 0.8 μl of 4-μM BODIPY 505/515 (Fam et al. 2018) in dimethyl sulfoxide (DMSO) (Thermo Fisher) were added to the Eppendorf tube containing the cell suspension. The tube was mixed for 1 min using a vortex to ensure proper mixing of the staining reagents. Following mixing, the tube was placed in the dark and incubated for 5 min. After incubation, cells were subjected to another round of centrifugation for 5 min at 13 000 g. The resulting pellet was resuspended in 0.8 ml of saline solution. A microscopy slide was prepared by adding 10 μl of the cell suspension, and fluorescence was visualized with a fluorescent microscope (Axiocam 202) at 500 nm (BODIPY) and 350 nm (CFW).

### Lipidomic analysis

#### Sample preparation

*Malassezia* spp. were cultured for 7 days on mDixon plates in a 33°C incubator (BINDER) with high humidity. After 7 days, FA-starved cultures were made by adding 3-ml *Malassezia* cells to 7.5-ml 4x MMM and 19.5-ml Milli-Q in a 100-ml Erlenmeyer flask and incubating in a 33°C incubator (New Brunswick Scientific) at 180 rpm for 3 days. Subsequently, 3-ml FA-starved cells were transferred to a new 100-ml Erlenmeyer flask with 7.5-ml 4x MMM, 16.5-ml Milli-Q supplemented with 3-ml 5-mM FA, and 10% NP-40 Tergitol™ (final concentration 0.5-mM FA and 1% NP-40 Tergitol™). After another 4 days (first growth step), 10-ml cells were taken and mixed in a 250-ml Erlenmeyer flask with 25-ml fresh 4x MMM, 55-ml Milli-Q and supplemented with 10-ml 5-mM FA and 10% NP-40 Tergitol™ and cultured for another 3 days at 33°C at 180 rpm (second growth step). The same procedure was followed for cultures involving ox bile, but the MMM was supplemented with 20-mg l^−1^ ox bile (Carlroth) in 10% NP-40 Tergitol™(final concentration 2-mg l^−1^ ox bile and 1% NP-40 Tergitol™). In case of culturing in mDixon, FA-starved cells were immediately transferred to 100-ml mDixon and grown for 4 days at 33°C at 180 rpm. When OD600 values reached between 0.5 and 1.0, cells were harvested. Liquid cultures were divided over 50-ml conical bottom tubes (Greiner) and spun down at 3000 g at 21°C for 10 min using an Eppendorf centrifuge at RT. The supernatant was discarded, and the pellet was washed 2 times with 5-ml PBS at RT. After washing 1-ml PBS was added to the pellet and resuspended. The samples were transferred to 1.5-ml Eppendorf tubes. Samples were placed in Techne Dri-block DB 2A at 60°C for 15 min to inactivate the cells. Subsequently, they were centrifuged at 6000 g for 10 min using an Eppendorf centrifuge. The supernatant was discarded, and samples were lyophilized for 24 h (ilShinBioBase, Scala Scientific). After lyophilization, the samples were weighed and stored at −20°C.

### Lipid extractions

Lipid extraction was carried out as described by Matyash et al. (2008), with specific modifications by Metabolomics Core Facility (MetCore), Universidad de los Andes, Bogotá, Colombia (https://metcore.uniandes.edu.co/es/) as described. Methanol (MeOH) was added to each sample, followed by vigorous vortexing. Subsequently, methyl tert-butyl ether was added to the mixture, and the samples were vortexed once more. Finally, water was included, and the samples were centrifuged at 14 000 g for 5 min. Lipidomic analysis, quality assurance, quality control, and data analysis were performed at Metabolomics Core Facility (MetCore).

The samples were analyzed using an Agilent Infinity 1260 liquid chromatography system coupled to an Agilent 6545 quadrupole time-of-flight mass spectrometer analyzer with electrospray ionization (Agilent Jet Stream ESI source). 1 μl of the sample was injected into an Agilent InfinityLab Poroshell 120-EC-C18 column (3.0 × 100 mm, 2.7-Micron; Agilent, USA) at 60°C. The mobile phase employed was composed of 60:40 ACN:H_2_O with 10-mM ammonium formate and 0.1% formic acid (Phase A) and a mixture of 90:10 IPA:ACN H2O with 10-mM ammonium formate and 0.1% formic acid (Phase B) with a constant flow rate of 0.6 ml/min. The elution gradient was programmed with the following specifications: 0 min 15% (B), 0–2 min 30% (B), 2–2.5 min 48% (B), 2.5–11 min 82% (B), 11–11.5 min 99% (B), 11.5–12 min 99% (B), 12–12.1 min 15% (B), and 12.1–18 min 15% (B). The mass spectrometer was operated in positive and negative ESI ionization modes separately, with a range of 65 to 1700 m/z. The QTOF instrument was operated in 4-GHz (high resolution) mode. Capillary voltage was set to 3500, the drying gas flow rate was 8 L/min at 325°C, gas nebulizer 35 psi, fragmentor voltage 120 V, skimmer 65 V, and octopole radio frequency voltage (OCT RF Vpp) 750 V. Data were collected in centroid mode at a scan rate of 3 spectra per second. Two reference masses, m/z 121.0509 [purine, (C5H4N4 + H)+] and m/z 922.0098 [HP-0921, (C18H18O6N3P3F24 + H)+] were used for mass correction in positive mode throughout the analysis.

Quality assurance and quality control (QA/QC) procedures adhered to established guidelines to minimize undesirable variations (Kirwan et al. 2022). To ensure the cleanliness of equipment, materials, and reagents used in sample preparation, solvent blanks, and extraction blanks were analyzed at the start of each sequence. A pooled QC sample was prepared by combining equal portions from every extract using the same process for lipidomic analyses. This QC was injected ten times at the outset of the run and then after every eight samples. Moreover, the biological samples were randomly arranged within the sequence to mitigate any potential biases.

The data were manually processed and inspected using Agilent Mass Hunter Profinder 10.0 software. Data obtained from alignment, deconvolution, and integration were first normalized by total area and subsequently subjected to filtering based on criteria including blank values, reproducibility, and presence. For the blank filter, features present in a sample with an intensity at least 10 times greater than the blank was retained. For reproducibility filtering, the coefficient of variation (CV, %) of the area was calculated in the QC samples, and features with a CV of more than 20% were excluded. Subsequently, for presence filtering, data had to be present in a minimum of 80% of the samples in at least one group to be retained.

### Statistical analysis

Multivariate statistical analysis (MVA) was employed using SIMCA-P + 17.0 (Umetrics) and MetaboAnalyst (https://www.metaboanalyst.ca/) to identify the lipids with statistically significant differences between groups. PCA was employed as an unsupervised method to assess the data quality. Following that, supervised Partial Least Squares Discriminant Analysis (PLS-DA) was performed for precise classification or prediction in specific groups, along with supervised Orthogonal Partial Least Squares Discriminant Analysis (OPLS-DA) models to determine the molecular attributes responsible for the group separation. The models were applied with unit variance (UV) scaling.

Statistical analysis and data graphing were carried out using Metaboanalyst (https://www.metaboanalyst.ca/) and the R packages ggplot2, readr, dplyr, and tidyr. ANOVA was used to compare groups, and a *P*-value of < 0.05 indicated statistical significance. As a first step in this annotation process, statistically significant molecular features, as determined by the MVA analysis (specifically, those with a Variance Important in Projection (VIP) > 2), were subjected to annotation. Lipids were annotated using MS-DIAL 5.1.230912 (https://systemsomicslab.github.io/compms/msdial/main.html), SIRIUS 5 (https://boecker-lab.github.io/docs.sirius.github.io/), and metabolic databases KEGG (https://www.genome.jp/kegg/) and Metacyc (https://www.genome.jp/kegg/), and Lipid MAPS (https://www.lipidmaps.org). This annotation process involved evaluating the isotopic pattern distribution within the molecular formula derived from the experimental data and considering the potential formation of adducts using the CEU Mass Mediator (http://ceumass.eps.uspceu.es/mediator/). Additionally, the exact observed mass of each compound was compared with databases, including METLIN (https://metlin.scripps.edu/), KEGG (https://www.genome.jp/kegg/), Lipid MAPS (https://lipidmaps.org/, https://www.lipidmaps.org), and HMDB (https://hmdb.ca/). This annotation process involved evaluating the isotopic pattern distribution within the molecular formula derived from the experimental data and considering the potential formation of adducts using the CEU Mass Mediator (http://ceumass.eps.uspceu.es/mediator/). Additionally, the exact observed mass of each compound was compared with databases, including METLIN (https://metlin.scripps.edu/), KEGG (https://www.genome.jp/kegg/), Lipid MAPS (https://lipidmaps.org/), and HMDB (https://hmdb.ca/).

## Results

### No growth of *Malassezia* spp. in defined medium with FFA in the presence of Brij-58

**Figure 1.**
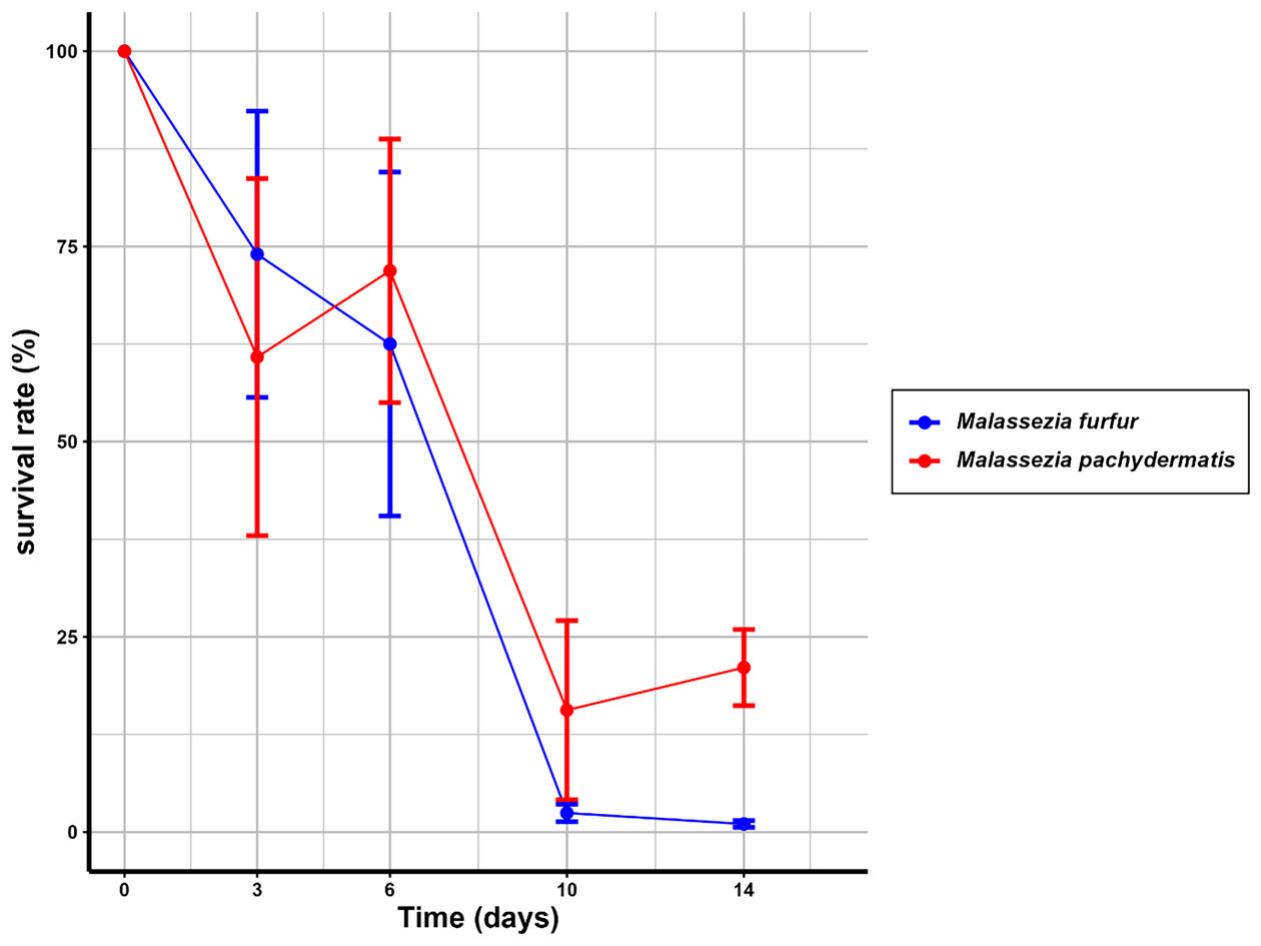
Viability of *Malassezia* spp., after incubation in MMM without FFA. Viability of *Malassezia* spp., after incubation in MMM without FFA and detergents at 33°C. Samples were plated on mDixon agar plates at the indicated time points to determine the CFU. Survival (%) is expressed as a fraction of the total amount of cells determined at the start of the experiment. The experiment was repeated four times, and the SEM was determined.

**Figure 2.**
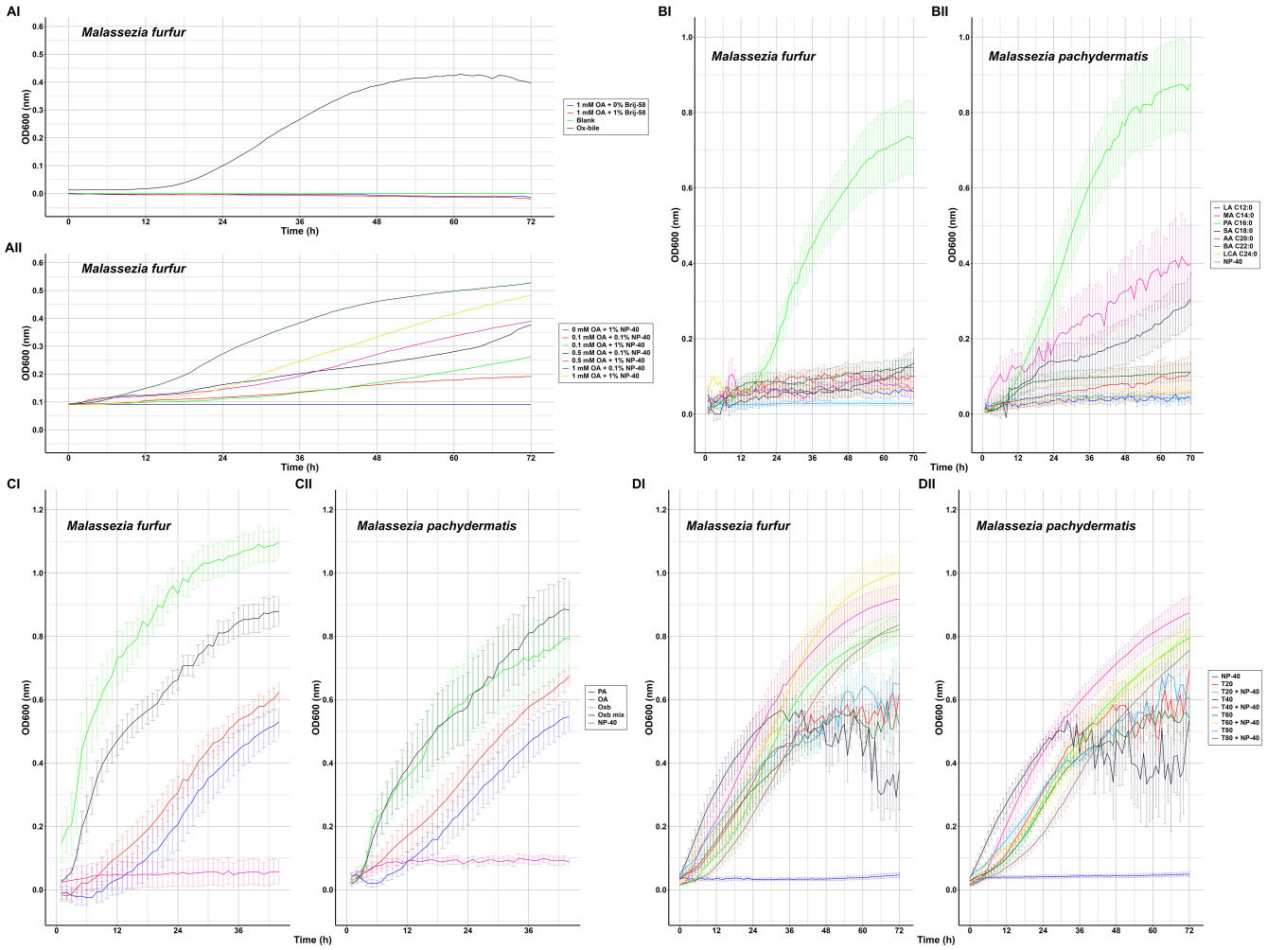
Growth analysis of *Malassezia* spp in defined medium. Growth of FA-starved *M. furfur* in de MMM with 1-mm OA in the absence or presence of 1% Brij-58 (AI) or varying concentrations (0.1, 0.5, or 1 mM) of OA in the absence or presence of 0.1 or 1% NP-40 Tergitol™ (AII). Data are the average of two experiments. Growth of *M. furfur* (BI) *and M. pachydermatis* (BII) in MMM in the presence of 1 mM of the indicated FFA with 1% NP-40 Tergitol™ at 33°C. No growth was observed in NP-40 Tergitol™ lacking FFA. Experiments were performed at least as triplo and the average OD600 nm with SEM was determined. Growth of *M. furfur* (CI) *and M. pachydermatis* (CII) in MMM in the presence of 1 mM of PA, OA, or a mixture of 0.5-mM PA and 0.5-mM OA with 1% NP-40 Tergitol™ at 33°C. Growth was compared to growth in defined medium with ox bile (2 g/L final) or in ox bile mix (ox bile 2 g/L, 0.2-ml/L OA, and 1-ml/L Tween 40 final concentrations) in the presence of 1% NP-40 Tergitol™. No growth was observed in NP-40 Tergitol™ lacking FFA. Experiments were performed at least as triplo, and the average OD600 nm with SEM was determined. Growth of *M. furfur* (DI) *and M. pachydermatis* (DII) in defined medium with the indicated Tween variants in the absence or presence of 1% NP-40 Tergitol™. Experiments (each a triplicate) were repeated two times, and the average OD600 nm with SEM was determined.

Previously, we analyzed the growth of different *Malassezia* spp. in a defined medium with FFAs in the presence of 1% Brij-58 (w/v) and observed that growth was inefficient (Triana et al. 2017). The growth observed may be due to the carry-over of lipids and Tween 80 that were used to resuspend the cells from mDixon to MMM containing FFA and 1% Brij-58. We, therefore, decided to include a 3-day starvation phase at 33°C in MMM lacking FAs and Tween to reduce the carry-over of lipids. We first determined the survival of *M. furfur* and *M. pachydermatis* under such conditions since an *S. cerevisiae fas1 ole1* mutant was previously reported to lose viability very fast when deprived of FAs and detergent (Henry 1973). To that end, cells were incubated in MMM lacking FA and Tween for up to 14 days, and at regular intervals, the viability was determined by plating on mDixon and culturing at 33°C (Fig. 1). We observed a relatively slow decrease in viability of both *Malassezia* species under these conditions, with a half-life of ∼6 to 8 days at 33°C, much longer than previously observed for a *S. cerevisiae fas1 ole1* mutant. We therefore preincubated the *M. furfur* for 3 days, and these FA-starved cells were subsequently used in growth experiments in 96 wells of F-bottom plates with MMM supplemented with either oleic acid in the absence or presence of 1% Brij-58 and compared to growth in mDixon. However, no growth of *M. furfur* was observed in this defined medium, whereas growth was resumed in mDixon within 24 h (Fig. 2 AI). Similar results were observed for *M. pachydermatis* (not shown). We varied the concentration of Brij-58 in these experiments from 1%, 0.1%, 0.1%, or 0.001% in the presence of OA or PA at 1 mM, but under all these conditions, no growth was observed with either of these species (results not shown).

### Growth of *Malassezia* spp. in defined medium with FFA in the presence of NP-40 Tergitol™

Previously, the growth of the *S. cerevisiae fas1 ole1* mutant was observed in a defined medium with FFA and NP-40 Tergitol™ (Henry 1973). When FA-starved *M. furfur* was cultured in MMM with different concentrations of OA and 0.1 or 1% NP-40 Tergitol™, we observed growth (Fig. 2 AII). The efficiency of growth increased with the concentration of OA and NP-40 Tergitol™ presence, and no growth was observed in the absence of OA but in the presence of 1% NP-40 Tergitol™. We subsequently analyzed the *Malassezia* spp. preference for specific FFA and determined growth in presence of 1-mM saturated FFA with increasing chain length (LA, MA, PA, SA, AA, BA or LCA) in the presence of 1% NP-40 Tergitol™ (Fig. 2B). Both species could grow most efficiently with PA (C16:0), while only limited growth was observed for *M. furfur* with the other FFA tested (Fig. 2 BI). Growth of *M. pachydermatis* was also observed with SA (C 18:0) and AA (C:20), although less efficient than with PA, while only limited growth was observed with the other FFA tested (Fig. 2 BII). Growth was confirmed by microscopic analysis of the wells in which especially longer chain FFA (C16 and longer) also displayed some aggregation which was especially visible in the absence of cells (Supplementary Fig. S2).

The growth of *Malassezia* spp. in defined medium with 1-mM PA or 1-mM OA (C18:1) in the presence of 1% NP-40 Tergitol™ was investigated. In addition, we analyzed growth in a defined medium with ox bile or an ox bile mix—containing oxbile, OA, and Tween 40, which are major lipid sources in mDixon, in the presence of 1% NP-40 Tergitol™(Fig. 2C). Growth of both species with OA was comparable to growth with PA. Growth of both species was better in the complex lipid mixtures of ox bile and the ox bile mix as compared to growth with single FFA, and *M. furfur* was growing faster than *M. pachydermatis*. Ox bile supported the growth of *M. furfur* even better (Fig. 2 CI) as compared to the ox bile mix, while the growth of *M. pachydermatis* was similar in ox bile or ox bile mix (Fig. 2 CII).

### Growth of *Malassezia* spp. in the defined medium in the presence of Tweens

The non-ionic detergents Tween 20, Tween 40, Tween 60, and Tween 80 are frequently used to differentiate *Malassezia* spp. Both *Malassezia* spp. showed efficient growth in the presence of either Tween 20, Tween 40, Tween 60, or Tween 80 (Fig. 2D; T20, T40, T60, T80) in the presence of 1% NP-40 Tergitol™. Interestingly, in the absence of 1% NP-40 Tergitol™ growth curves did not increase further after approximately 30 h and showed a large variation in OD600. This variation is due to cell aggregation, which was prevented by NP-40 Tergitol™ (Fig. S3). Growth curves increased up to 72 h in the presence of NP-40 Tergitol™, and each Tween variant demonstrated efficient growth, but some differences in the efficiency were observed. *Malassezia furfur* might have a slight preference for T60 (Fig. 2 DI), whereas *M. pachydermatis* had a slightly higher preference for T40 in the presence of 1% NP-40 Tergitol™ (Fig. 2 DII).

### FFA preference of *S. cerevisiae fas1/fas2* mutant in defined medium and 1% NP-40 Tergitol™

**Figure 3.**
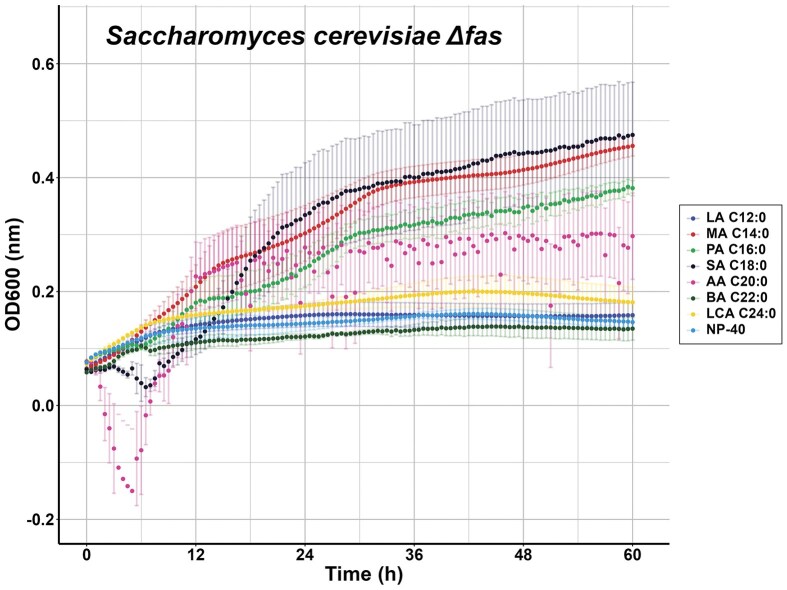
Growth of *S. cerevisiae fas1 fas2* mutant in defined medium. Growth of *S. cerevisiae fas1/fas2* (ΔFAS) mutant in MMM with 1% NP-40 Tergitol™ with the indicated FFA at 33°C. Some residual growth was observed with NP-40 Tergitol™ only. Experiments (each a triplicate) were repeated two times, and the average OD600 nm with SEM was determined.

We determined the FFA preference of *S. cerevisiae* mutant with inactivated *fas1* and *fas2* genes due to the insertion of the geneticin resistance gene. We used the MMM supplemented with amino acids and uracil to complement the auxotrophic markers of the strain. *Saccharomyces cerevisiae fas1/fas2* mutant was pre-grown in YPD medium and transferred to MMM supplemented with FFA and 1% NP-40 Tergitol™(Fig. 3). Interestingly, some residual growth was observed in the absence of FFA but presence of 1% NP-40 Tergitol™ which is most likely due to the carry-over of some lipids from the YPD medium. MA supported efficient growth of the *S. cerevisiae fas1/fas2*, whereas growth on PA was slightly less. We observe some growth in the presence of SA (C18:0) and, to a lesser extent, AA (C20:0) or LCA (C24:0), the other FA tested did not support growth. We observed a higher variability with some of the FFA tested, amongst others, SA (C18:0) and AA (C20:0). Microscopic analysis indicated the presence of FA aggregates visible in especially wells without cells, which were more prominently observed with PA, SA, and AA (Supplementary Fig. S4) and which were used for background measurements, thereby affecting OD measurements.

### Lipidomic analysis

**Figure 4.**
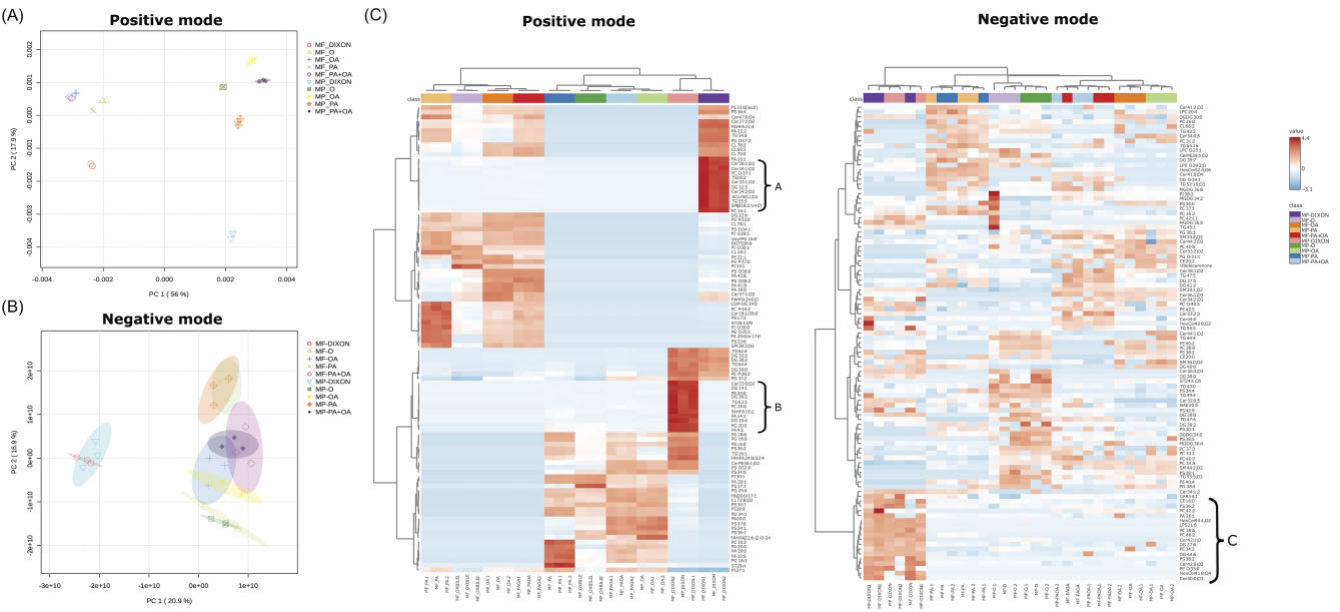
Lipidomic analysis of Malassezia spp. PCA analysis of lipidomes obtained in positive (A) or negative mode (B) of *M. furfur*(MF) *or M. pachydermatis* (MP) grown in mDixon (DIXON) or in MMM with 1% NP-40 Tergitol™ with either ox bile (o), oleic acid (OA), palmitic acid (PA), or palmitic and oleic acid (PA + OA). Heatmaps of lipidomes (C) obtained in the positive or negative mode of *M. furfur* (MF) or *M. pachydermatis* (MP) grown in mDixon (DIXON) or in MMM with 1% NP-40 Tergitol™ with either ox bile (o), oleic acid (OA), palmitic acid (PA), or palmitic and oleic acid (PA + OA). Indicated are group A, B, and C representing lipid species enriched especially in mDixon cultures.

**Figure 5.**
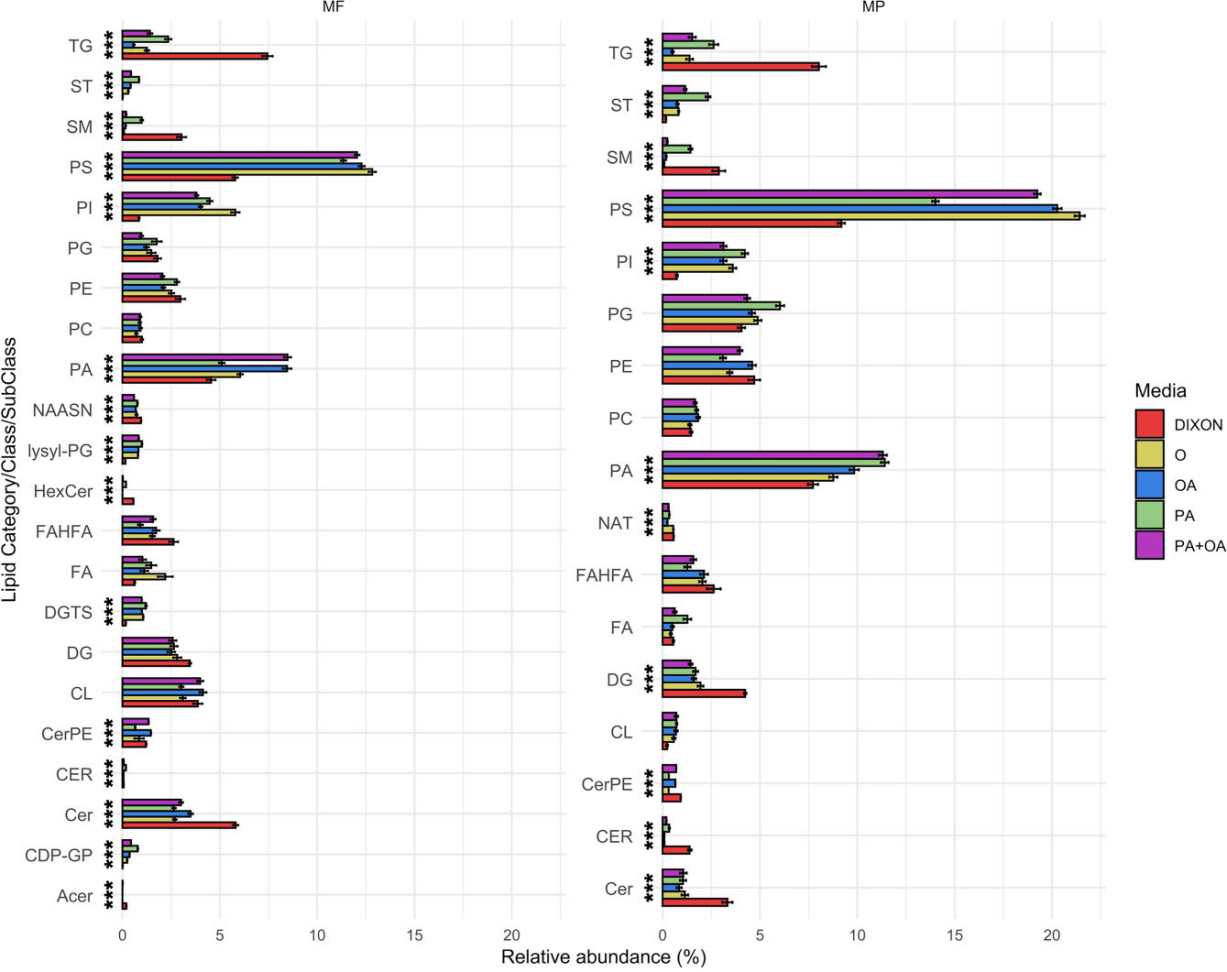

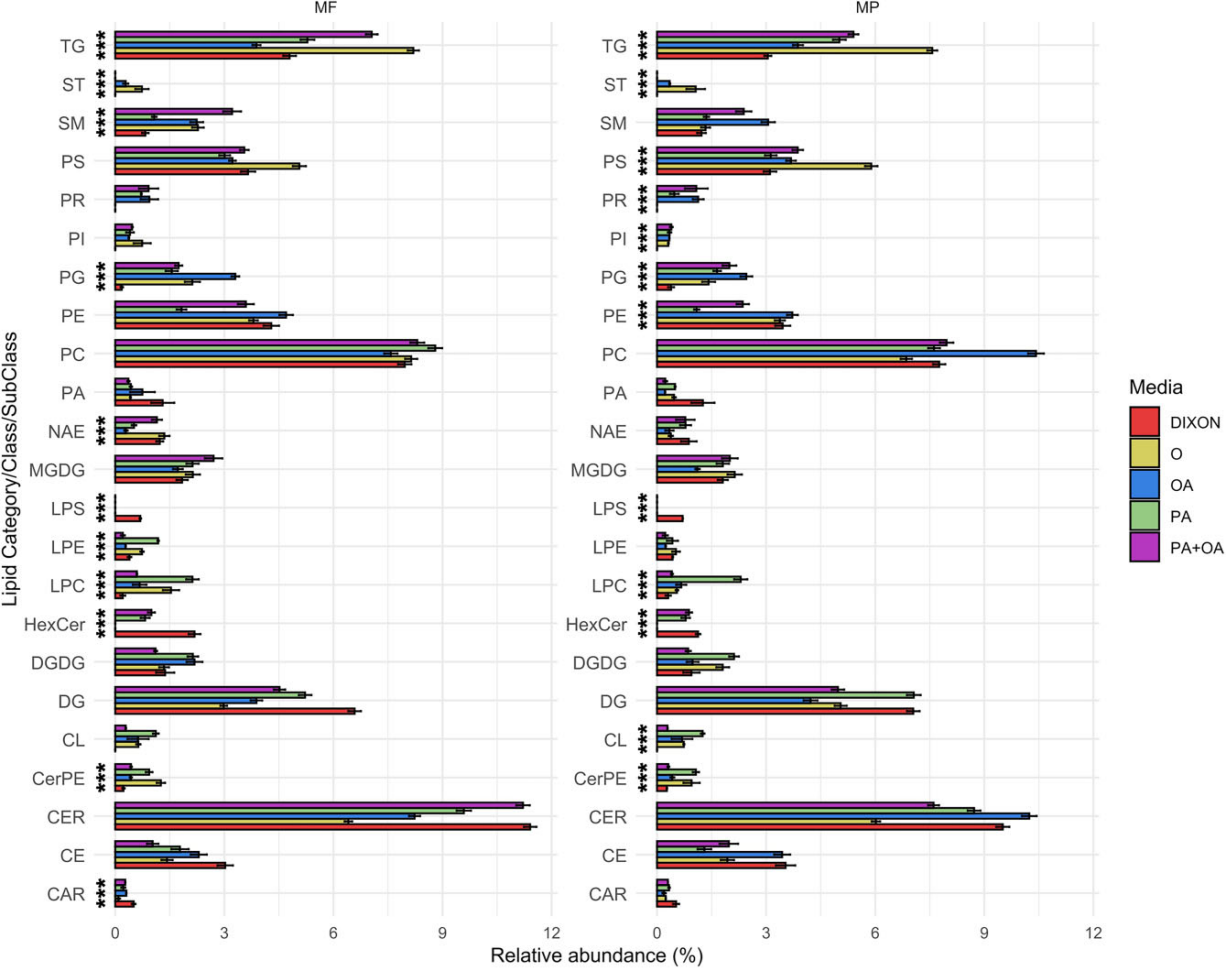
Relative ratios of lipid classes in the lipidome. Relative ratio of lipid classes detected in positive mode (A) or negative mode (B) in the lipidomes of *M. furfur* (MF, left) or *M. pachydermatis* (MP, right) grown in mDixon (DIXON) or in MMM with 1% NP-40 Tergitol™ with either ox bile (o), oleic acid (OA), palmitic acid (PA), or palmitic and oleic acid (PA + OA).The names of the lipid classes are described in Table S1. ***P-value < 0.001.

Total cell lipid extracts were prepared from three culture samples in MMM with FA and NP-40 Tergitol™ and from mDixon cultures. In positive mode and negative mode, 1209 and 1244 features were detected, respectively, with 160 and 101 lipid species being statistically different between groups and therefore they were annotated. PCA analysis differentiated the lipid composition of both species cultured in mDixon from those grown in each of the MMM cultures with 1% NP-40 Tergitol™ in both positive and negative mode (Fig. 4A and B). Interestingly, the lipid composition of both species grown in MMM with 1% NP-40 Tergitol™ and ox bile, a major lipid source in mDixon, was different as compared to the lipid compositions of mDixon-grown cells (Fig. 4A). Also, the lipid composition of cells grown in MMM with FFA and 1% NP-40 Tergitol™ differed considerably between both species in positive mode. Furthermore, the lipidomes of cells of *M. pachydermatis* grown in MMM and NP-40 Tergitol™ with ox bile, PA, OA or PA and OA were all different. This was not the case for *M. furfur*, where the lipidomes of cells grown in MMM with OA were more like PA and OA grown cells, while the lipidomes of cells grown in MMM with ox bile and PA were more alike (Fig. 4A, positive mode). In negative mode, the lipid compositions of each species grown in MMM with NP-40 Tergitol™ and one of the lipid sources were much more alike (Fig. 4B, negative mode). These differences in lipid composition were also visualized in the heatmaps of each of the samples (Fig. 4C). Lipidomes of cells grown in mDixon differed considerably, and a subset of enriched lipid species was detected in both positive and negative (Fig. 4C indicated with A, B, and C). Lipid species enriched in group A belonged amongst others to phosphatidyl choline (PC), ceramides (CER), sphingolipid species (SM), and TGs (Fig. S5 and Table S1). Group B lipids were enriched amongst others with various phospholipid species (PL), diglycerides (DG), and TG, while group C encompassed a variety of PL species (Fig. S5 and Table S1). Analysis of the relative abundance of lipid classes in positive mode indicated that the lipidome of both species grown in mDixon was significantly enriched in TG, SM, and CER (Fig. 5A and Table S1). Neutral lipids like DG and TG are stored in lipid droplets, and staining of lipid droplets with BODIPY showed approximately 10-fold more lipid droplets in cells grown in mDixon as compared to those grown in MMM with PA and NP-40 Tergitol™ (Fig. S6). FFAs were also detected, and *M. furfur* grown in mDixon was enriched for PA, while *M. pachydermatis* grown in mDixon was enriched for physeteric acid (C14:1) and fumaric acid (C4:2) (Fig. S7). In contrast, *M. pachydermatis* grown in MMM with 1% NP-40 Tergitol™ and FFA or ox bile was enriched for docosapentaenoic acid (C22:5) while *M. furfur* was enriched for halogenated FA (Dembitsky and Srebnik 2002). Interestingly, although in a low abundance (∼2.5%), betaine lipids diacylglyceryltrimethylhomo-Ser (DGTS) were found in *M. furfur* and were not found in *M. pachydermatis*. In particular, these lipids were substantially more abundant in cells grown with MMM with PA, OA, or ox bile with NP-40 Tergitol™ than in mDixon medium (Figs. 4C and 5).

## Discussion

Efficient growth of *Malassezia* spp. in defined medium with FFA can be instrumental to understanding lipid metabolism in these lipophilic fungal species. Here, we describe efficient FA-dependent growth of two *Malassezia* species in defined medium with a single FFA in the presence of NP-40 Tergitol™. This method can be exploited to study the FA-dependent growth of other recently described lipophilic fungal species (Nev et al. 2024), as well as species in which FA synthases are inactivated (Fischer et al. 2020). We show efficient growth of these two *Malassazia* spp. with PA (C16:0) or OA (C18:1), while *M. pachydermatis* also showed growth with SA (C18:0) or AA (C20:0) although to a lesser extent. Growth in defined medium with ox bile or ox-bile mix, representing the lipid-rich source in mDixon, both strongly supported growth in the presence of NP-40 Tergitol™. This can be explained by the presence of a larger variety of lipid and FA sources than media with a single FFA. Remarkably, the growth of both *Malassezia* spp. is relatively inefficient on MA (C14:0) in contrast to the *S. cerevisiae* Δfas1/Δfas2 mutant. We previously compared the genetic differences for genes encoding lipid biosynthesis enzymes between *S. cerevisiae* and *Malassezia* spp. and provided a general overview of lipid metabolism in *Malassezia*, based on lipidomic and *in silico* genomic analysis (Celis Ramirez et al. 2020). However, it is unclear why these species differ in their preference for MA in these culture conditions. This might be related to efficiency for uptake of MA, which might be less for *Malassezia* spp. tested, but more research is required to unravel this. Interestingly, growth on Tween 20 is very efficient, and Tween 20 contains, especially, LA (C12:0) and MA and, to a lesser extent, PA, SA, and OA (Fig. S1). Whether growth is better supported if FAs are presented in a lipid structure (like in Tweens or phospholipids) cannot be ruled out. We analyzed the growth of both *Malassezia* spp. in the presence of dioleoylphosphatidylcholine (containing only OA), dipalmitoylphosphatidylcholine (containing only PA), and dimyristoylphosphatidylcholine (DMPC, containing only MA) in 1% NP-40 Tergitol™ and found that each of these phospholipid species can indeed support growth (results not shown). FA-dependent growth therefore appears to be influenced by the way the FAs are presented, but more research is required to unravel this observation.

Growth in the different variants of Tween resulted in cell aggregation, which was previously observed while examining the cell wall of *M. furfur* (Mittag 1995). This study noted the attachment of neighboring cells by fusion of the laminar layer in Tween 80 supplemented culture, using electron microscopy. In 2009, it was demonstrated that Tween 80 grown cells exhibited withered surfaces and a thinner, more electron transparent cell wall compared to untreated *M. furfur* cells (Kim et al. 2009), indicating that detergents affect the outer cell structures, which might affect cell-cell aggregation.

Lipidome analysis underscores that *Malassezia* spp. can process FFA further into a large variety of FA species which can, e.g. be observed in the 20 most abundant lipid species in the lipidome detected in positive mode (Fig. S8). Growth of *M. pachydermatis* in MMM with PA (C16:0) results in the formation of lipid species with a large variety in FA length (e.g. PI28:0; PS34:0; PS37:0; or PG25:4). After the uptake of FFA and conversion to FA-CoA, the FA species can be elongated and converted to single or multiple unsaturated forms (Tehlivets et al. 2007). FA length reduction was also detected, e.g. *M pachydermatis* grown with OA (C18:1) produces TG16:1 or PS16:0 (Fig. S8) and alpha-oxidation of methyl- or hydroxy-branched FA to odd chain length might be involved (Mori et al. 2023). Moreover, a variety of phospholipid species were detected after lipidome analysis. Since we used complete cells for lipid extraction, part of the phospholipids detected can be expected to originate from organelles, e.g. PE and CL, and some lipid intermediates are synthesized explicitly in mitochondria (Malina et al. 2018). FA synthesis in mitochondria proceeds via the bacterial type II fatty acid synthase (FAS II) enzyme as described for yeast. In yeast, octanoyl-ACP (C8:0-ACP) is synthesized via this acyl-carrier-dependent mechanism, a precursor for lipoic acid synthesis, a co-factor for a subset of mitochondrial enzymes (Schonauer et al. 2009). Interestingly, in the mitochondria of mammalian cells, the mitochondrial FAS (mtFAS) was also shown to synthesize longer acyl chains of at least C14. Still, these FAs appeared to remain attached to the ACP (Angerer et al. 2017, Nowinski et al. 2020). Whether *Malassezia* spp. have adapted the mtFAS to a system to produce a subset of FA is unclear and requires further analysis but could also explain the presence of some shorter chain FA in the lipidome. Clearly, mtFAS cannot complement the absence of cytosolic FAS, which produces C16 and C18-CoA as end products in yeast (Gajewski et al. 2017), since growth in the absence of exogenous FAs is not observed.

Remarkably, both *Malassezia* spp. prefer C16:0 (PA), and their growth is comparable with C18:1 (OA) in this system. In contrast, the *S. cerevisiae Δfas1/Δfas2* mutant was able to grow with MA (C14:0) most efficiently. Although uptake and activation of MA may be possible via long-chain fatty acyl-CoA synthetases detected in the genome of *Malassezia* spp. (Celis Ramirez et al. 2020), growth on MA as individual FFA appears less efficient. It should be noted that both *Malassezia* spp. did grow efficiently in MMM when MA was provided via DMPC and 1% NP-40 Tergitol™ (data not shown). Also, the growth of both *Malassezia* spp. is efficient with Tween 20 (Fig. 2D) which contains especially shorter FAs C12:0, C14:0 and C14:1 (Fig. S1). These results suggest that growth of *Malassezia* spp. is especially influenced by the way FAs are presented to cells and that uptake of MA can be efficient if it is presented as a phospholipid or a detergent. Further analysis is required to determine if MA as FFA cannot reach the FA-uptake systems in both *Malassezia* spp. in contrast to yeast. Furthermore, transcriptomic and/or proteomic analysis are required to investigate if the expression of FA-uptake systems of *Malassezia* spp. is influenced by the way FAs are presented to *Malassezia* cells.

We observed the presence of DG in the lipidome of both species. DG can be produced after the hydrolysis of phospholipids or TG and are intermediates in the synthesis of TG, which occurs in the endoplasmic reticulum and lipid droplets in yeast (Sorger and Daum 2003) and growth in mDixon resulted in the formation of neutral lipids, which accumulate in lipid droplets (Mantilla et al. 2021). The amount of lipid droplets is markedly lower after the growth of both *Malassezia* spp. in a defined medium with PA and NP-40 Tergitol™ as compared to growth in mDixon. Even growth in ox bile did not result in a lipidome comparable to the cells grown in mDixon. We previously determined the lipidome of different *Malassezia* spp. after growth in mDixon, and we could not exclude the possibility that lipids from mDixon remained associated with the cells (Celis Ramirez et al. 2020). Here, we cultured *Malassezia spp*. after a FA-starvation period in a defined medium, and repeated growth in at least two steps in a defined medium, which must have reduced possible associated lipids to a large extent. Growth in the defined medium is a better procedure to follow lipid metabolism, which can e.g. be supplemented with tracer-labeled FA species or (labeled) lipid intermediates to determine the uptake and/or *de novo* synthesis of lipid species in *Malassezia* spp. We observed that both *Malassezia* spp. survived FA starvation in the defined medium for a longer time as was previously reported for *S. cerevisiae fas1* or *fas1/ole1* mutant (Henry 1973). These latter mutants were shown to undergo logarithmic cell death during FA starvation. We propose that *Malassezia* spp. have adapted in some way to survive extended periods of FA starvation, which could occur in their natural habitat. Skin is very limited in nutritional sources, and lipids in sebum can be an important source for microorganisms living on the skin (Swaney et al. 2023). Competition between microbial species might vary the availability of lipid sources and require adaptation. Interestingly, DGTS lipid was detected only in *M. furfur* after growth in MMM with FA or ox bile. These betaine-derived lipids are a metabolic response to phosphate starvation conditions and used in the phospholipid remodeling of the cell membrane (Bhalla et al. 2022). This difference in lipid composition could potentially contribute to the distinct physiological characteristics and ecological niches occupied by these two *Malassezia* species as well as the availability of resources for these niches (Mayser et al. 1998). For instance, *M. furfur* is more commonly associated with PV and fungemia, while *M. pachydermatis* is frequently found as a commensal on animal skin and in cases of otitis external in dogs (Hobi et al. 2022). Moreover, the presence of DGTS in *M. furfur* might influence its membrane fluidity, permeability, or interaction with host cells, therefore affecting its pathogenicity as has been reported for other pathogenic yeasts (Ikeh et al. 2017, Lev et al. 2019, Kohler et al. 2020) and explained by the lipid metabolism flexibility reported for *Malassezia* (Triana et al. 2017). More research is required on how the survival of *Malassezia* spp., during FA starvation is regulated and which role DGTS lipids play in the adaptation of *Malassezia* spp. under stress.

## Supplementary Material

foaf043_Supplemental_File
